# A Rare Case of Pseudoxanthoma Elasticum Identified by Ocular Angioid Streaks

**DOI:** 10.7759/cureus.57342

**Published:** 2024-03-31

**Authors:** Iqra Mushtaq, Khushboo Goyal, Deepaswi Bhavsar, Renu Magdum

**Affiliations:** 1 Ophthalmology, Dr. D. Y. Patil Medical College, Hospital and Research Centre, Pune, IND

**Keywords:** optical coherence tomography, fundus camera photography, pseudoxanthoma elasticum, chicken skin, angioid streaks

## Abstract

A 42-year-old female with a known case of hypertension for three years, symptoms of metamorphopsia, and decreased vision in both eyes reported to the ophthalmology outpatient department. There was no recorded history of ocular injury or surgery. Several observational techniques, such as fundus inspection, fundus camera photography, and optical coherence tomography (OCT), were utilized to assess the patient. We referred her to the Department of Dermatology for additional assessment because of her symptoms as well as the appearance of her neck's skin, which matched "plucked chicken skin." There, the diagnosis of pseudoxanthoma elasticum (PE) was confirmed. She was subsequently scheduled for an intravitreal bevacizumab injection called Avastin, which improved her visual acuity.

## Introduction

Pseudoxanthoma elasticum (PE) is an autosomal recessive condition and is characterized by the gradual mineralization of calcium compounds in the connective tissues' elastic fibres, which has negative consequences on the eyes and other systemic signs. The typical finding of this disease in the fundus is angioid streaks which is a break in the bruch’s membrane [1].^ ^About 59-87% cases of angioid streaks are due to PE. Atrophic lesions of the retinal pigment epithelium (RPE), crystalline bodies, optic disc drusen, choroidal neovascularization, subretinal hemorrhage, and fibrosis are other ocular symptoms [2]. Other ocular abnormalities include "peau d'orange" pigmentary alteration.

The most often associated conditions with angioid streaks are sickle-cell anemia, thalassemia, spherocytosis, as well as osteitis deformans (Paget's disease), Ehlers-Danlos syndrome, acromegaly, and Marfan syndrome. Apart from trauma, other conditions that have been linked to angioid streaks include alpha-beta lipoproteinemia, acquired hemolytic anemia, hemochromatosis, hypertension, diabetes, etc. [3].

The angioid streaks are often brittle, thickened, and calcified Bruch's membrane dehiscence accompanied by overlaying RPE atrophy. They are present in a circumferential pattern of grey or dark red linear lesions with irregularly serrated edges that surround the optic disc and spread outward from the peripapillary region. Typically, they are bilateral. Most angioid streak instances eventually progress to visual impairment. In this instance, we used bevacizumab intravitreal injections to prevent secondary choroidal neovascularization (CNV) of PE from occurring [4].

## Case presentation

**Figure 1:**
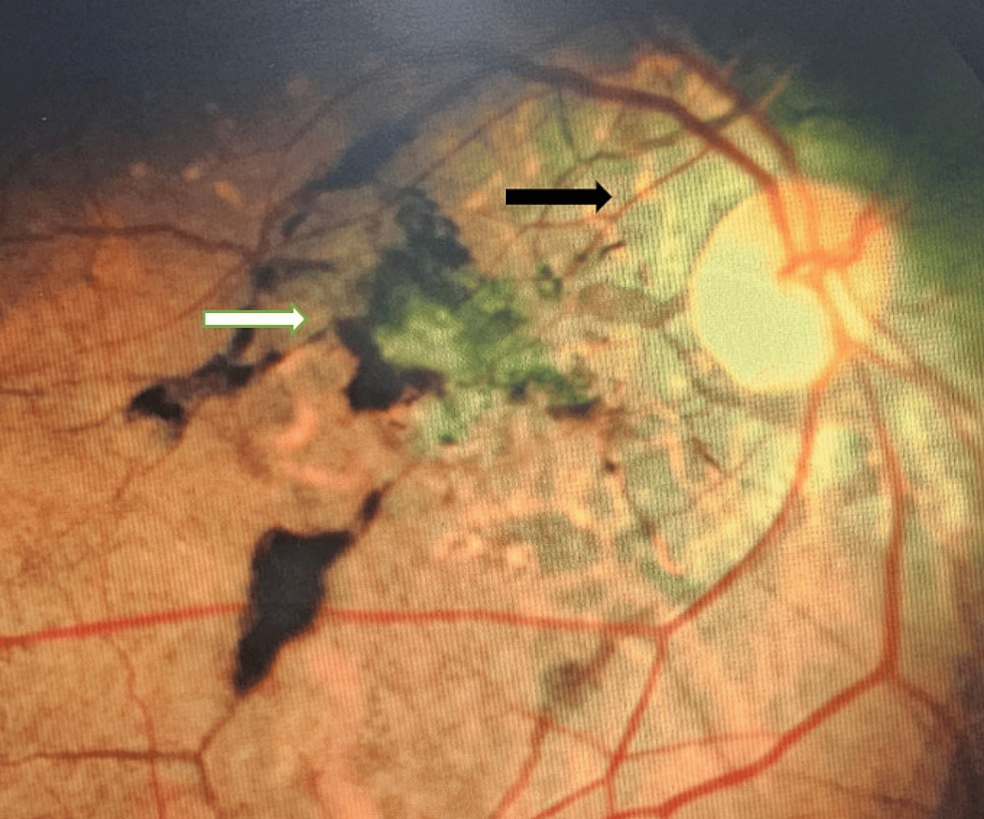
Photomicrograph of right eye (RE) fundus showing scarred choroidal neovascular membrane (CNVM) (white arrow ) with angioid streaks (black arrow).

**Figure 2:**
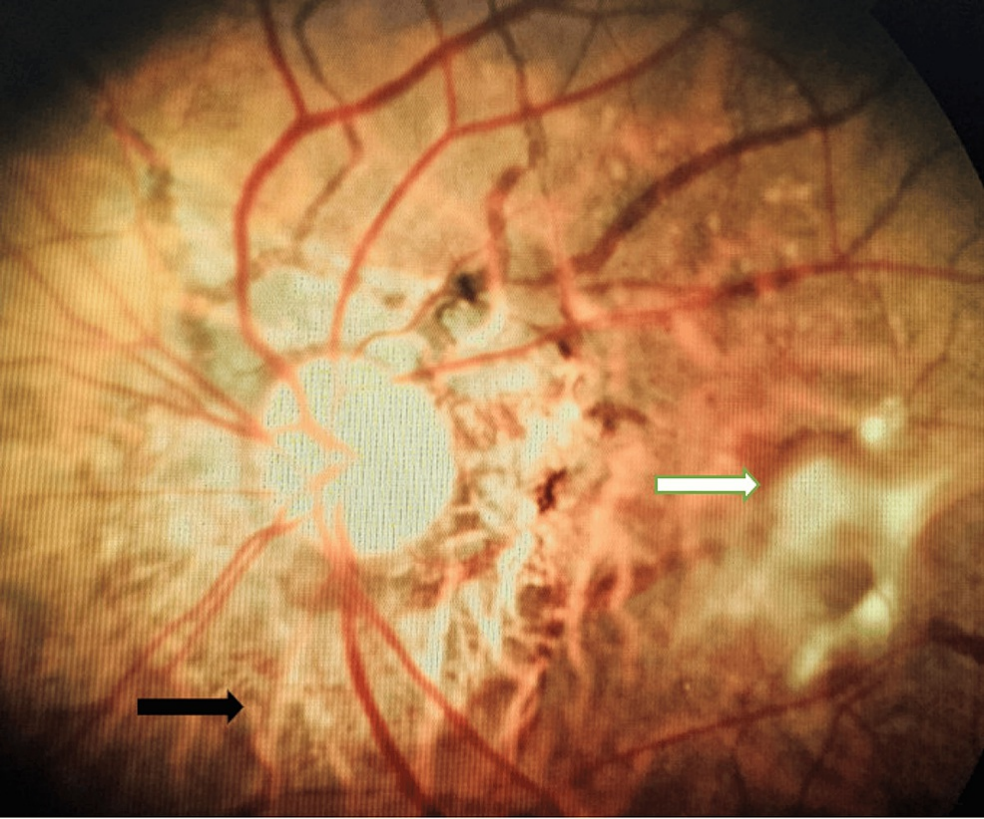
Photomicrograph of left eye (LE) fundus showing active choroidal neovascular membrane (CNVM) (white arrow) with angioid streaks (black arrow).

A 42-year-old female came with problems of metamorphopsia and a decrease in vision in both eyes (left eye more than right eye) in the last two months. The patient was a known case of hypertension for the last three years and with no significant history of any ocular injury or surgery in the past. The patient denied a history of any similar complaints in the family. The best corrected visual acuity (BCVA) of both eyes was 6/60 on Snellen’s visual acuity chart. On eye examination, the right eye's uncorrected distance visual acuity was 6/60 which did not improve with any refraction, while the left eye's BCVA was 6/60 (-1.50, -1.00 at 90). IOP measurements on applanation tonometry for the right and left eyes were 14 and 16 mmHg, respectively. There were no notable results in either eye's anterior segment examination. Upon fundus examination, both eyes' optic discs were found to have radial angioid streaks (AS), as well as scarring over the macula in the right eye (RE) and choroidal neovascular membrane in the left eye (LE) (Figures 1, 2)

**Figure 3:**
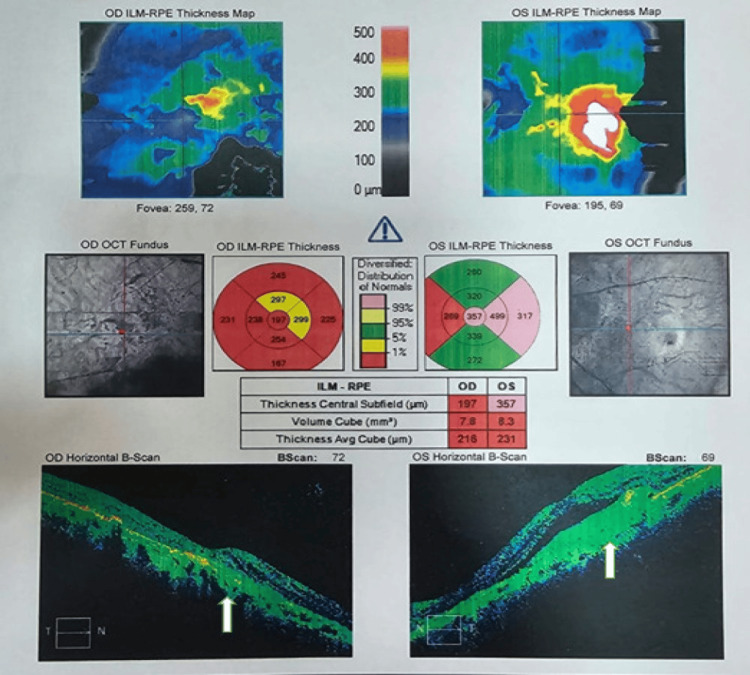
Figure showing optical coherence tomography (OCT) showing choroidal neovascularization in both eyes and breaks in the retinal pigment epithelium (RPE) (white arrow). Intraretinal fluid is seen along with foveal thinning.

Optical coherence tomography (OCT) showed choroidal neovascularization in both eyes and breaks in the RPE were evident on the OCT (Figure 3).

**Figure 4:**
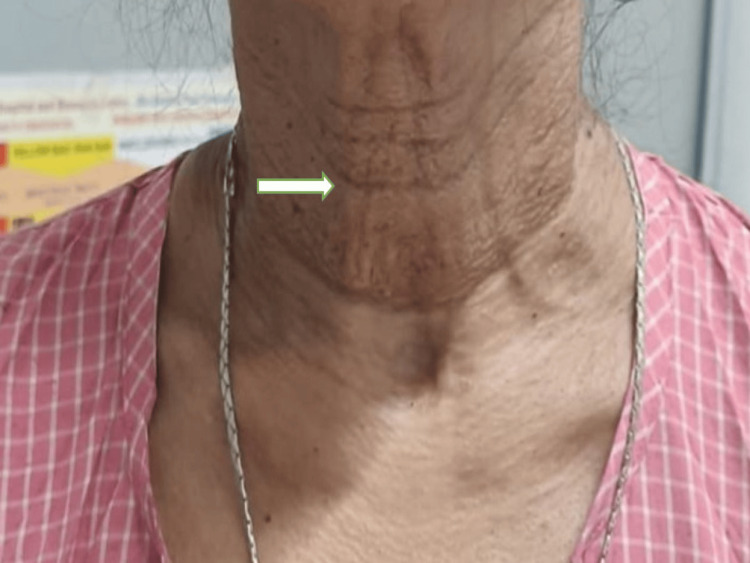
Picture showing characteristic plucked chicken skin appearance of the neck (white arrow).

On general examination, the characteristic texture of the skin of her neck resembled "plucked chicken skin" (Figure 4). For a potential PE diagnosis, the patient was sent to the dermatology division.

After ruling out surgical contraindications, she received the first dosage of intravitreal injection (anti-VEGF) of bevacizumab (Avastin) in the left eye to control the progression of choroidal neovascularization (CNV). After two weeks of intravitreal injection, her vision in the left eye improved to 6/36 and the CNV lesion area was gradually shrinking. She was planned for a further course of Avastin injections at regular intervals.

## Discussion

An inherited condition known as PE causes progressive calcification and degeneration of elastic fibres. The most common inheritance pattern is autosomal recessive, though autosomal dominant variants are also seen. Skin, cardiovascular, and oracular problems are its outward signs. Yellowish papules that form around the neck, under the arms, on the inside of the elbows, on the backs of the knees, and on the groins are frequent signs of skin complaints. These papules give the patient's skin a "plucked chicken skin" appearance. The illness often develops slowly and has a late beginning [5].

The severe ocular manifestations which can lead to blindness are usually seen at the later stages of the disease. Angioid streaks are radial streaks that radiate from the optic disc to both eyes' equatorial borders. and are colored red, brown, or grey. They are comparable in length in both eyes and get longer over time. The calcification and rupture of Bruch's membrane are the primary pathologies of angioid streaks. The backbone supporting the integrity of the basement membrane (BM) is a multi-layered, lattice-like fibrous structure called the elastin layer (EL). Angioid streak formation is caused internally by the calcification and deterioration of EL [6].

Retinal pigment epithelium secretes vascular endothelial growth factor (VEGF) which reaches the choroid since the Bruch's membrane is ruptured. This has an impact on choroidal blood flow, which causes a reduction in intrinsic choroidal capillary blood flow, which results in nutritional deficiency, toxin buildup, inflammation, and subsequent ischaemia. As a result, a neovascular membrane develops and can expand through Bruch's membrane gap between the RPE and photoreceptor layers. Since the lesion affects the macula, the haemorrhage and extravasation cause dramatic vision loss. This was controlled by the use of anti-VEGF injection of Avastin which helped in reducing the exudation and bleeding.

## Conclusions

Here, we present a case of a patient whose best corrected visual acuity (BCVA) improved following anti-vascular endothelial growth factor (anti-VEGF) medication. Rarely do typical pseudoxanthoma elasticum (PE) patients present with both cutaneous and ocular symptoms. We were able to make a diagnosis on the basis of fundus changes and dermatological examination. Also, anti-VEGF injections were able to effectively treat the choroidal neovascularization that developed because of angioid streaks of PE.
